# Gastric Intestinal Metaplasia in Children and Adolescents Is Reversible upon Reaching Adulthood—Results from a Long-Term Cohort Study

**DOI:** 10.3390/cancers17010128

**Published:** 2025-01-03

**Authors:** Jan Drnovšek, Nina Zidar, Jera Jeruc, Lojze M. Šmid, Gaj Vidmar, Borut Štabuc, Matjaž Homan

**Affiliations:** 1 Department of Gastroenterology, University Medical Centre Ljubljana, Japljeva ulica 2, 1000 Ljubljana, Slovenia; jan.drnovsek@kclj.si (J.D.);; 2 Faculty of Medicine, University of Ljubljana, Vrazov trg 2, 1000 Ljubljana, Slovenia; 3 Institute of Pathology, Faculty of Medicine, University of Ljubljana, Korytkova ulica 2, 1000 Ljubljana, Slovenia; 4 Department of Biostatistics and Scientific Informatics, University Rehabilitation Institute, Linhartova cesta 51, 1000 Ljubljana, Slovenia; 5 Faculty of Mathematics, Natural Sciences and Information Technologies, University of Primorska, Glagoljaška cesta 8, 6000 Koper, Slovenia; 6 Department of Gastroenterology, Hepatology and Nutrition, University Children’s Hospital, Bohoričeva ulica 20, 1000 Ljubljana, Slovenia

**Keywords:** gastric intestinal metaplasia, preneoplastic lesion, gastric cancer, *Helicobacter pylori*, paediatric, esophagogastroduodenoscopy

## Abstract

Background/Objectives: Gastric intestinal metaplasia (GIM) is considered an irreversible preneoplastic precursor for gastric adenocarcinoma in adults. However, its significance in children and the long-term outcome remain poorly understood. Methods: All children diagnosed with GIM between 2000 and 2020 were identified at a large tertiary referral centre. Upon reaching adulthood (≥18 years), the patients were invited to undergo follow-up esophagogastroduodenoscopy (using narrow-band imaging additionally to high-definition white light endoscopy), with gastric biopsies obtained according to the updated Sydney protocol. Childhood and adulthood gastric biopsies were re-evaluated by two experienced gastrointestinal pathologists using Kreyberg staining. Results: Paediatric GIM was diagnosed in 178/14,409 (1.2%) esophagogastroduodenoscopies performed during the study period. Fifty adult patients with childhood GIM agreed to participate in the study. The mean age at childhood and adulthood endoscopies were 14.3 years (median 15) and 25.2 years (median 24), respectively. The mean follow-up interval was 10.5 years. All childhood GIM cases were classified as complete-type. Notably, GIM completely resolved in 41/50 of patients (82%) by the time of adulthood follow-up. No dysplasia or carcinoma was detected in any patient. Childhood *Helicobacter pylori* infection, similar to other evaluated host-related factors, was not significantly associated with the persistence of GIM into adulthood (11.2% vs. 29.3%, *p* = 0.41). Conclusions: Childhood GIM was a rare finding but demonstrated a high rate of reversibility by adulthood regardless of *Helicobacter pylori* status, with no cases of dysplasia or carcinoma observed during long-term follow-up.

## Simple Summary

Gastric intestinal metaplasia (GIM) is regarded as “the point of no return” in the gastric cancerogenesis sequence. *Helicobacter pylori* infection remains the leading etiologic factor for non-cardia gastric cancer in adults. On the other hand, the causes and evolution of GIM in children remain poorly understood. The aim of this study was to evaluate the long-term outcome of GIM once children reached adulthood, after a mean follow-up of a decade between both esophagogastroduodenoscopies with gastric sampling. Furthermore, the results of our study provide valuable insights into the risk factors and surveillance considerations of GIM, diagnosed in children. The results suggest the possibility of discontinuing surveillance for limited complete-type GIM diagnosed in childhood, in patients without additional risk factors for gastric adenocarcinoma.

## 1. Introduction

Gastric intestinal metaplasia (GIM) is a histopathologic condition defined by the replacement of the normal gastric epithelium with intestinal-type columnar containing goblet cells, with or without Paneth cells and absorptive cells [1]. The Correa cascade, a widely accepted model for the pathogenesis of gastric cancer, proposes that chronic inflammation induces chronic gastritis, which progresses to atrophic gastritis (AG), GIM, dysplasia, and ultimately, gastric cancer [2]. Chronic infection with *Helicobacter pylori* (*H. pylori*) remains the leading etiologic factor for non-cardia gastric cancer in adults [3,4,5,6].

While AG often improves following *H. pylori* eradication [7], the occurrence of GIM has traditionally been considered “the point of no return” in the gastric cancerogenesis sequence [2]. Nevertheless, *H. pylori* eradication may reduce the risk of gastric cancer [8,9]. Beyond *H. pylori* infection, host-related risk factors for GIM development have been extensively studied in the context of gastric cancer pathogenesis. These factors include age, male sex, ethnicity, blood group, family history of gastric cancer, bile reflux, tobacco smoking, excessive alcohol consumption, and dietary habits [10,11,12,13,14,15,16]. Interestingly, the prevailing view of GIM as an irreversible precancerous lesion in adults has been challenged in recent years [17,18].

On the other hand, the evidence regarding GIM in the paediatric population remains scarce. While GIM can be detected in gastric biopsies of children undergoing esophagogastroduodenoscopy (EGD) regardless of the indication, data on its prevalence, natural history, and clinical relevance are limited. The few available studies, often with small sample sizes, have yielded conflicting results [19,20,21,22]. A recent metanalysis by Kalach et al. reported a GIM prevalence of in 4.2% in antral mucosa and 1.3% in corpus mucosa samples with *H. pylori*- infected children [23].

Due to the scarcity of data, the long-term outcome of GIM diagnosed in childhood remains poorly understood. To our knowledge, there is no consensus on optimal management or surveillance strategies for paediatric GIM, as the natural progression of the condition remains unclear. To address this knowledge gap, we conducted a longitudinal cohort study to assess the long-term outcome of GIM in patients who were initially diagnosed in childhood and followed into adulthood.

## 2. Materials and Methods

### 2.1. Identification of the Patients

A retrospective search of the electronic pathology database at the University Children’s Hospital Ljubljana, Slovenia, was conducted to identify all paediatric patients (<18 years old) with histologically confirmed GIM in gastric biopsies obtained during EGD between January 2000 and December 2020, regardless of the indication for EGD. The electronic medical records were reviewed to extract patient demographic and clinical information, including age at index EGD, *H. pylori* status, comorbidities, and medical treatment history.

### 2.2. Recruitment of the Patients

Patients meeting the inclusion criteria were invited to undergo follow-up EGD via two consecutive telephone calls. The inclusion criteria were (1) histologically confirmed GIM on initial EGD, (2) age ≥18 years at the time of study enrolment, (3) absence of major comorbidities, (4) capacity to provide informed consent, and (5) willingness to participate in the study. The exclusion criteria were (1) insufficient biopsy specimens collected at the initial examination, (2) inadequate histological material quality precluding reliable GIM diagnosis, (3) medical contraindications to sedation for EGD, and (4) pregnancy.

Eligible patients who consented to participate in the study completed a study questionnaire inquiring about their family history of gastric cancer in first- or second-degree relatives, blood group, tobacco and alcohol use, antibiotic usage for any indication, history of *H. pylori* eradication therapy, long-term use of proton pump inhibitors (PPIs), number of additional EGDs after the initial procedure, and dietary habits, including salt intake, consumption of spicy foods, and daily intake of processed foods.

### 2.3. Endoscopy

Follow-up EGDs were performed by two experienced endoscopists using Olympus Exera III or Fujifilm EG-760 series endoscopes. Adequate mucosal visualisation was ensured through sufficient carbon dioxide insufflation, gastric mucosal cleaning, and a minimum examination time of 7 min. The greater and lesser curvatures, antrum, pylorus, incisura, fundus, and cardia were systematically inspected and photographically documented. In addition to high-definition white light endoscopy, narrow-band imaging was utilised to enhance the visualisation of potential precancerous lesions. Gastric biopsies were obtained according to the updated Sydney protocol, including a minimum of two biopsies from the antrum (within 2–3 cm of the pylorus from both lesser and greater curvatures), one from the incisura angularis, and two from the corpus (one from the lesser curvature approximately 4 cm proximal to the angle and one from the greater curvature approximately 8 cm distal to the cardia). All biopsies were collected using standard 2.8 mm Olympus Endo Jaw FB-240-U forceps and stored in formalin. Cassettes were labelled to ensure accurate anatomical identification. Additional oesophageal, gastric, or duodenal biopsies were obtained if indicated by endoscopic findings.

### 2.4. Histopathological Assessment

The biopsy samples were fixed in formalin, embedded in paraffin, and stained with haematoxylin and eosin (HE), Giemsa, and the modified Kreyberg method. All original and follow-up gastric biopsy specimens were independently evaluated by two experienced gastrointestinal pathologists at the Institute of Pathology, Faculty of Medicine at the University of Ljubljana, Slovenia, in accordance with established international guidelines [1,24].

Gastric intestinal metaplasia was defined as the replacement of the normal gastric epithelium with intestinal-type epithelium. GIM was classified as “complete” when both Paneth and goblet cells were present in the intestinal epithelium and “incomplete” in the absence of Paneth cells. The distribution of GIM was categorised as “limited” if confined to the antrum or incisura and “extensive” if involving both the antrum and corpus or the corpus alone.

**Figure 1:**
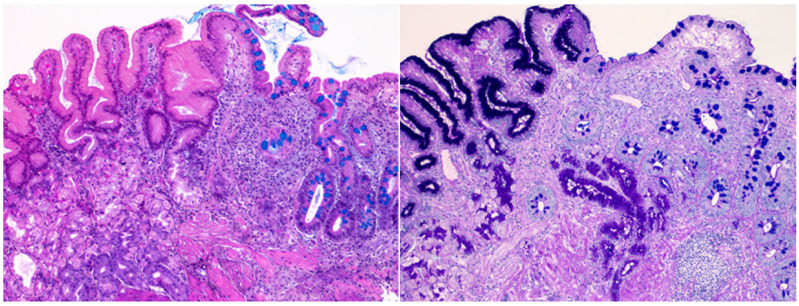
Intestinal metaplasia, complete type. (**Left**): Kreyberg staining demonstrates blue cytoplasmic mucin vacuoles in the area of intestinal metaplasia, contrasting with the absence of blue staining in the preserved gastric mucosa. (**Right**): Alcian Blue-Periodic acid Schiff staining shows blue-stained cytoplasmic mucin vacuoles in the area with intestinal metaplasia, along with strong staining of the brush border in the area of preserved gastric mucosa. Original magnification 10×.

For this study, biopsy samples were additionally stained with Alcian Blue-Periodic acid Schiff (AB-PAS) and re-evaluated by two expert pathologists to confirm the GIM type, distribution, and morphometry. GIM was diagnosed and typed using both AB-PAS and Kreyberg staining. Kreyberg staining proved superior for delineating the GIM areas (Figure 1), and thus, the Kreyberg-stained slides were utilised for morphometric analysis. Morphometry was performed using NIS Elements Microscope Imaging Software version 4.30 (Nikon, Tokyo, Japan). This analysis involved measuring the total biopsy sample area (in square micrometres) and the area occupied by GIM (in square micrometres) per vial. The proportion of mucosa occupied by GIM was then calculated and reported as a percentage.

### 2.5. Statistical Analysis

Descriptive statistics were calculated for numerical variables, with distribution visualised using histograms. For binary descriptive variables, confidence intervals (95% confidence interval, CI) for the proportions were estimated using the Wilson method. Given the small sample size, unreliable data on risk factors, and the problem of multiple testing, the association of individual risk factors with GIM was assessed using only descriptive statistics. The association of the presence of multiple risk factors with GIM was analysed using a logistic-regression model, with the number of risk factors as the sole predictor. Firth’s correction was applied to account for the small sample size. A *p*-value less than 0.05 was considered statistically significant.

## 3. Results

### 3.1. Patients Enrolment Process

We identified 178 paediatric patients who had undergone EGD with gastric biopsies of any indication at the University Children’s Hospital Ljubljana, Slovenia, between January 2000 and December 2020, whose stomach biopsies revealed the presence of GIM. Of these, 153 were eligible for study participation based on age (≥18 years). However, due to the extended interval between the initial EGD and the study period, contact could not be established with 72 (47%) patients. An additional 16 (10%) patients declined to participate. Patients with significant comorbidities or pregnancy were also excluded from the study.

**Figure 2:**
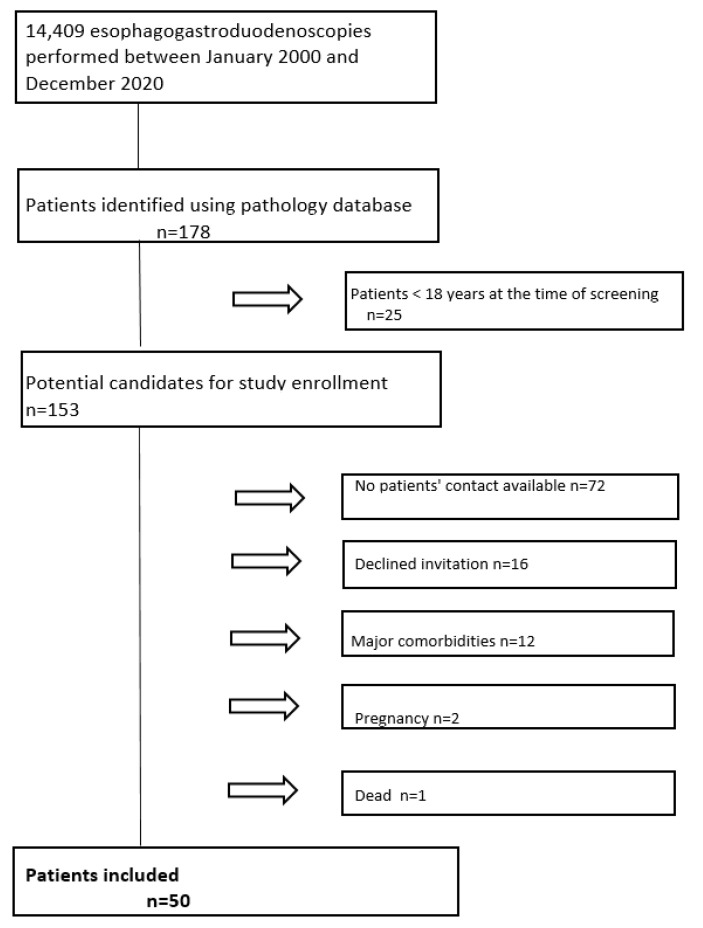
Flowchart of the patient selection process.

Finally, follow-up EGD with gastric sampling was performed on 50 healthy individuals (Figure 2).

### 3.2. Estimated Prevalence of Gastric Intestinal Metaplasia

During the study period, 14,409 EGDs with gastric biopsies were performed in children and adolescents in our centre. Accordingly, we estimated a prevalence of paediatric GIM of 1.2% (178/14,409) in our study population.

### 3.3. Patient Characteristics

All patients were Caucasian, and 30/50 (60%) were female. The mean age at initial EGD was 14.3 years (median 15 years), and the mean age at follow-up EGD was 25.2 years (median 24 years). The mean interval between EGDs was 10.5 years (median 10 years, range 2–22 years).

### 3.4. Endoscopic and Histopathological Findings

The most common indications for the initial EGD in childhood were abdominal pain, vomiting, and failure to thrive. Endoscopic features of gastritis were found in 23/50 patients (46%), reflux esophagitis in 10/50 (20%), Crohn’s disease in 6/50 (12%), coeliac disease in 3/50 (6%) and eosinophilic esophagitis in 2/50 patients (4%). In six patients (12%), the endoscopic mucosal appearance was normal. Histopathologically, all patients with GIM also exhibited chronic gastritis (active or inactive). However, the extent of GIM (limited or extensive) could not be determined due to the biopsy protocols in place at the time, which did not include separate sampling from the antrum and corpus. In total, 14 out of 50 patients (28%) had concomitant AG with GIM in their initial biopsies. All patients presented with complete-type GIM at the time of initial EGD in childhood. Notably, no patient presented with isolated GIM without additional pathological findings in the gastric biopsies.

At the follow-up EGD in adulthood, the most common endoscopic finding was gastric mucosal erythema (27/50 patients, 54%). Despite the use of narrow-band imaging, no suspicious focal lesions were visualised. Histopathologically, chronic gastritis was found in 35/50 (70%) patients, while isolated AG was observed in 2/50 patients (4%). Persistent GIM was identified in only 9/50 (18%) patients at follow-up. Among these patients, six had coexisting AG and GIM, while three had GIM with chronic gastritis but without AG. All persistent GIM cases were of the complete type and, except for one patient, limited to the antrum. Notably, GIM completely resolved in 41/50 (82%) patients. No dysplasia or carcinoma was detected in any patient. The proportion of mucosa occupied by GIM per biopsy ranged from 2.05% to 19.98% (mean 5.3%).

Furthermore, no cases of gastric carcinoma were recorded in the National Cancer Registry for any of the 178 patients initially diagnosed with GIM, including those who did not participate in the follow-up study (Figure 2).

### 3.5. Helicobacter Pylori Infection

**Figure 3:**
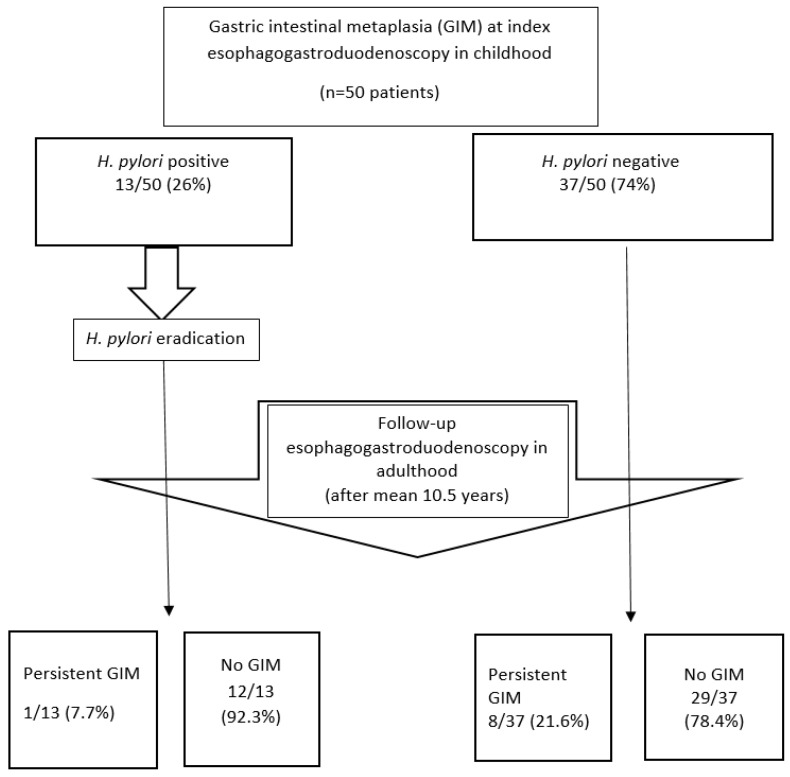
Resolution of childhood gastric intestinal metaplasia stratified by *H. pylori* infection at diagnosis.

Among patients with GIM in our cohort, *H. pylori* infection was found in 13/50 patients (26%; 95% CI: [16–40%]) at the initial EGD. According to paediatric guidelines, patients received either standard clarithromycin-based triple therapy or antibiotic susceptibility testing-guided triple therapy, with a urea breath test performed at least 4 weeks post-treatment to confirm eradication [25]. Out of 13 patients with *H. pylori* infection at initial EGD, only one had persistent GIM at the follow-up EGD. In the remaining 12/13 (92.3%) patients, GIM was not detected at follow-up (Figure 3).

At the follow-up EGD, *H. pylori* infection was detected in 6 individuals (12%; 95% CI: [6–24%]), none of whom had GIM in their gastric biopsies. Additionally, 4 patients (8%; 95% CI: [3–19%]) had *H. pylori* infection across both, the initial and follow-up EGDs, with a mean interval of 14.7 years (range 10–22 years) between the two assessments. None of these patients had detectable GIM in their follow-up biopsies (Table 1).

### 3.6. Other Risk Factors for Gastric Intestinal Metaplasia

We evaluated eleven non-*H. pylori* related risk factors potentially associated with GIM development: family history of gastric cancer in first or second-degree relatives, blood group, tobacco smoking, excessive alcohol consumption, antibiotic use for any indication, multiple *H. pylori* eradication therapies, long-term proton pump inhibitor (PPI) use, at least one additional EGD after the initial procedure, heavily salted diet, consumption of spicy foods, and daily intake of processed foods containing nitrite and nitrate additives. Blood group data were not included in this analysis, as the data were only available for 22/50 (44%) patients. Patients presented with 0 to 4 of the remaining risk factors. The number of risk factors was not significantly associated with GIM persistence, as determined by a non-significant logistic regression model (likelihood ratio test: *p* = 0.92). Among the eight patients who had persistent GIM (data for one patient were not available), one had no risk factors, two had one risk factor, four had two risk factors, and one patient had three risk factors (Table 2).

## 4. Discussion

Our study provides valuable insights into the natural history, clinical significance, risk factors, and surveillance considerations for GIM in the paediatric population. In our cohort, paediatric GIM was rare and showed a favourable long-term outcome. The childhood GIM demonstrated a high rate of spontaneous resolution by adulthood regardless of *H. pylori* status, with no cases progressing to dysplasia or carcinoma.

The reported prevalence of GIM in children varies significantly across studies [20,22,26]. The GIM prevalence in our study cohort was 1.2%, which is lower than the reported prevalence in adult studies [27,28]. The result is not surprising, since exposure to risk factors over an extended period of time and aging are major risk factors for the development of GIM [29]. Consistent with our findings, a recent paediatric meta-analysis and systematic review found a GIM frequency of 1.2% in antral biopsies [23], while another study found a slightly lower frequency of 0.75% in the same location [21].

Gastric intestinal metaplasia has been traditionally considered an irreversible pathohistological “point of no return” in the carcinogenic cascade in adults [2]. However, the clinical importance and long-term outcome of GIM in childhood are poorly understood. Our study revealed that the majority (82%) of paediatric GIM cases resolved spontaneously a decade after initial diagnosis, and no cases progressed to dysplasia or carcinoma, even in those with persistent GIM. These findings challenge the notion of GIM’s irreversibility and suggest a more benign course in the paediatric population.

While *H. pylori* infection is strongly associated with an increased prevalence of GIM in adults and considered a pivotal step in gastric carcinogenesis, our findings suggest a less pronounced association in the paediatric population. GIM was rarely associated with *H. pylori* infection at index EGD, with most cases being *H. pylori*-negative (37/50, 74%). Moreover, only one out of 13 patients with *H. pylori* at index EGD exhibited persistent GIM at follow-up. Notably, none of the six individuals with *H. pylori* infection at follow-up EGD had GIM. Furthermore, in the subset of four (8%) patients with *H. pylori* infection across both EGDs, no GIM was detected in follow-up biopsies. However, it is important to note that PPI use at index EGD in almost one-third (28%) of patients may have influenced the *H. pylori* detection rate in our study. Shabib et al. [30] reported a significantly higher frequency of GIM in children with *H. pylori*-positive gastritis (42%) compared to those with *H. pylori*-negative gastritis (6%). Conversely, Kato et al. [31] found no difference in GIM prevalence between children with and without H. *pylori* infection. Additionally, no GIM was detected in a Brazilian cohort of 96 children with *H. pylori* gastritis [32], and a recent American study reported only a rare association between GIM and *H. pylori* gastritis [20]. In the recent metanalysis from Kalach et al., GIM was present in 4.2% of biopsy samples of antral mucosa and in 1.3% in corpus mucosa samples in children infected with *H. pylori* [23]. Notably, a Slovenian study of 165 *H. pylori*-infected children also found no increased incidence of precancerous gastric lesions [33].

Patients who have a first-degree relative with gastric cancer have an increased risk for GIM neoplastic progression [34,35]. However, GIM was not found in any of the four patients in our study with positive family history at the follow-up EGD. In addition, all our patients were Caucasians, which is a low-risk population for non-cardia gastric cancer [36,37].

Non-*H. pylori*-related host risk factors, including age, male sex, ethnicity, blood group, family history of gastric cancer, bile reflux, tobacco smoking, alcohol consumption, and diet, have been extensively studied in gastric cancer pathogenesis and are recognised as independent risk factors for the development of GIM [10,11,12,13,14,15,16,29]. In our study, we assessed the contribution of these factors to GIM development and its potential irreversibility. However, no correlation was found between these investigated risk factors and the natural course of GIM.

An unanswered question remains regarding the long-term management of GIM in children, particularly concerning surveillance after reaching adulthood. Unlike the adult population, for whom surveillance guidelines exist [38], no such recommendations have been established for paediatric patients. The results of our study suggest the possibility of discontinuing surveillance for limited complete-type GIM diagnosed in childhood, in patients without additional risk factors such as a positive family history of gastric cancer, autoimmune gastritis, or persistent *H. pylori* infection.

We acknowledge some limitations of our study. Foremost, the small sample size limits the generalisability of the results and the power of the statistical analysis. Clinicians should be aware of this consideration when interpreting results of these preliminary findings. The low prevalence of GIM that we observed may be attributed to sampling error, as GIM can be patchy, and its detection depends on the location and number of biopsies obtained. Gastric biopsies at the initial EGD were primarily taken from the antrum and not according to the updated Sydney protocol. Additionally, during patient enrolment, study candidates were identified solely as “GIM-positive” without specifying the anatomical location. Therefore, we were unable to define the distribution of GIM at the initial EGD or assess the correlation between the GIM extent and its histopathological outcome at follow-up. Finally, caution should be exercised in generalising our findings to other ethnicities, as our study population consisted exclusively of Caucasians.

## 5. Conclusions

In summary, GIM was a rare finding in childhood. In the majority of cases, it was reversible after reaching adulthood, contrasting with its typically irreversible nature in adults. Importantly, no clear correlation with *H. pylori* infection was observed in this study. Larger, multicentre prospective studies with standardised serial gastric sampling, comprehensive histological subtyping, and detailed risk factor assessment are needed to confirm these findings and establish definitive guidelines for the management and surveillance of GIM in the paediatric population.

## Figures and Tables

**Table 1:** Proportion of patients with persistent gastric intestinal metaplasia into adulthood stratified by *H. pylori* infection status from childhood to adulthood.

	Gastric Intestinal Metaplasia (GIM) —Evolution From Childhood Into Adulthood			
	Total Cohort (n = 50)	Persistent GIM (n = 9)	Resolved GIM (n = 41)	*p-*Value *
*H. pylori* infection				
Never	37/50 (74%)	8/9 (88.9%)	29/41 (70.7%)	0.41
Childhood only	13/50 (26%)	1/9 (11.1%)	12/41 (29.3%)	0.41
Adulthood only	2/50 (4%)	0/9 (0%)	2/41 (4.9%)	1.00
Childhood and adulthood	4/50 (8%)	0/9 (0%)	4/41 (9.9%)	1.00

* Chi-square/Fisher’s Exact test for persistent versus resolved gastric intestinal metaplasia.

**Table 2:** Presence of host-related risk factors at follow-up EGD in patients with GIM and without GIM.

			Presence of Paediatric Gastric Intestinal Metaplasia After Reaching Adulthood	
			No 41/49 * (83.7%)	Yes 8/49 * (16.3%)
**Risk Factors**				
Family history of gastric cancer			4 (9.8%)	0
Tobacco smoking			5 (12.2%)	1 (12.5%)
Alcohol consumption			11 (26.8%)	3 (37.5%)
Use of antibiotics for any indication			16 (39%)	2 (25%)
Multiple *H. pylori* eradication			1 (2.4%)	0
Long term treatment with PPI			12 (29.3%)	3 (37.5%)
At least one additional EGD after the initial			16 (39%)	4 (50%)
Heavily salted diet			3 (7.3%)	0
Strongly spiced food intake			1 (2.4%)	0
Daily consumption of industrially processed food			3 (7.3%)	0

Likelihood ratio test of logistic regression model for association of number of risk factors with GIM: *p* = 0.92. * Data were available for 49 out of the 50 patients.

## Data Availability

The data used for this report are available from the corresponding author upon reasonable request.
